# Supplementary material for: Synthesis and Pharmacological Evaluation of Hybrids Targeting Opioid and Neurokinin Receptors
Source: Molecules. 2019 Dec 5;24(24):4460. doi: 10.3390/molecules24244460 (PMC6943619; doi:10.3390/molecules24244460)
Supplement: Supplementary file 1 [file molecules-24-04460-s001.pdf]

Supporting information

Synthesis and pharmacological evaluation of hybrids targeting opioid and neurokinin receptors

Karol Wtorek[a], Anna Adamska-Bartłomiejczyk[a], Justyna Piekielna-Ciesielska[a], Federica Ferrari[b], Chiara Ruzza[b], Alicja Kluczyk[c], Joanna Piasecka-Zelga[d], Girolamo Calo'[b], Anna Janecka[a] *

[a]*Department of Biomolecular Chemistry, Medical University of Lodz, Lodz, Poland*

[b] *Department of Medical Sciences, Section of Pharmacology, University of Ferrara, 44121 Ferrara, Italy*

[c]*Faculty of Chemistry, University of Wroclaw, Wroclaw, Poland*

[d]*Institute of Occupational Medicine, Research Laboratory for Medicine and Veterinary Products in the GMP Head of Research Laboratory for Medicine and Veterinary Products, Lodz, Poland.*

Contents

Physicochemical characterization of analogs **2-7** (Table S1)…………………….………………..……p. S2

High resolution MS spectra (Fig. S1-S6) ……………………………..p. S3

Concentration-response curves of hybrid analogs **2-7** in the functional assay (Fig. S7) .... p. S10

Table S1. Physicochemical characterization of hybrids **2-7**.

No.	Sequence	Molecular Formula	Molecular Weight	m/z calcul. for $[M+2H]^{2+}$	m/z found for $[M+2H]^{2+}$
2	H-Tyr-[D-Lys-Phe-Phe-Asp]-Asn-D-Trp-Phe-D-Trp-Leu-Nle-NH_2	$C_{84}H_{102}N_{16}O_{14}$	1559.8075	780.3953	780.3904
3	H-Tyr-[D-Lys-Phe-Phe-Asp]-D-Trp-Phe-D-Trp-Leu-Nle-NH_2	$C_{80}H_{96}N_{14}O_{12}$	1445.7048	723.3739	723.3701
4	H-Tyr-[D-Lys-Phe-Phe-Asp]-Gln-Phe-Phe-Gly-Leu-Met-NH_2	$C_{73}H_{94}N_{14}O_{14}S$	1423.6779	712.3470	712.3422
5	H-Tyr-[D-Lys-Phe-Phe-Asp]-Phe-Phe-Gly-Leu-Met-NH_2	$C_{68}H_{86}N_{12}O_{12}S$	1295.5486	648.3177	648.3129
6	H-Tyr-[D-Lys-Phe-Phe-Asp]-Phe-Gly-Leu-Met-NH_2	$C_{59}H_{77}N_{11}O_{11}S$	1148.3748	574.7835	574.7780
7	H-Tyr-[D-Lys-Phe-Phe-Asp]-Gly-Leu-Met-NH_2	$C_{50}H_{68}N_{10}O_{10}S$	1001.2009	501.2493	501.2438

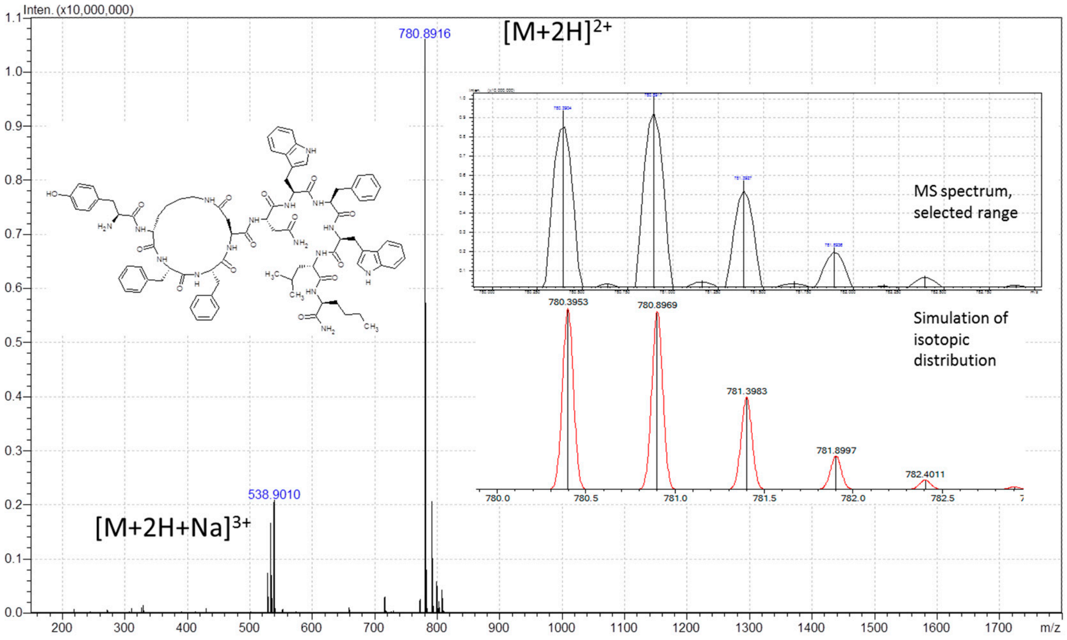

Figure S1. High resolution MS spectrum of peptide H-Tyr-(D-Lys-Phe-Phe-Asp)-Asn-D-Trp-Phe-D-Trp-Leu-Nle-NH_2 (analog 2). In inset, fragment of the experimental spectrum is compared with the simulated isotopic profile calculated for the expected molecular formula of protonated species $[M+2H]^{2+}$ (bottom panel).

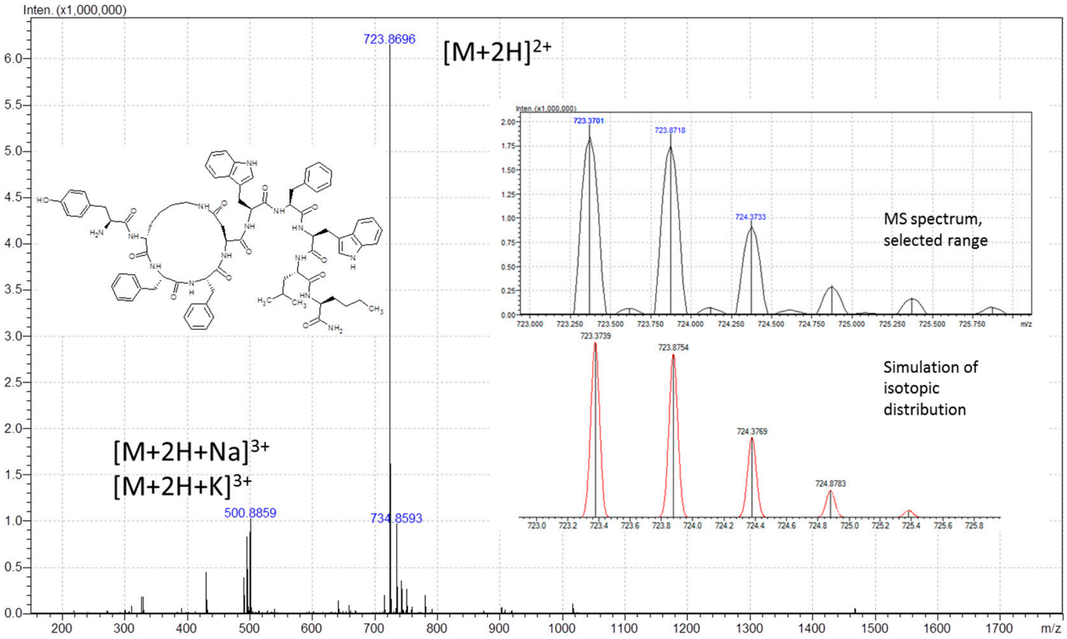

Figure S2. High resolution MS spectrum of peptide H-Tyr-(D-Lys-Phe-Phe-Asp)-D-Trp-Phe-D-Trp-Leu-Nle-NH_2 (analog 3). In inset, fragment of the experimental spectrum is compared with the simulated isotopic profile calculated for the expected molecular formula of protonated species $[M+2H]^{2+}$ (bottom panel).

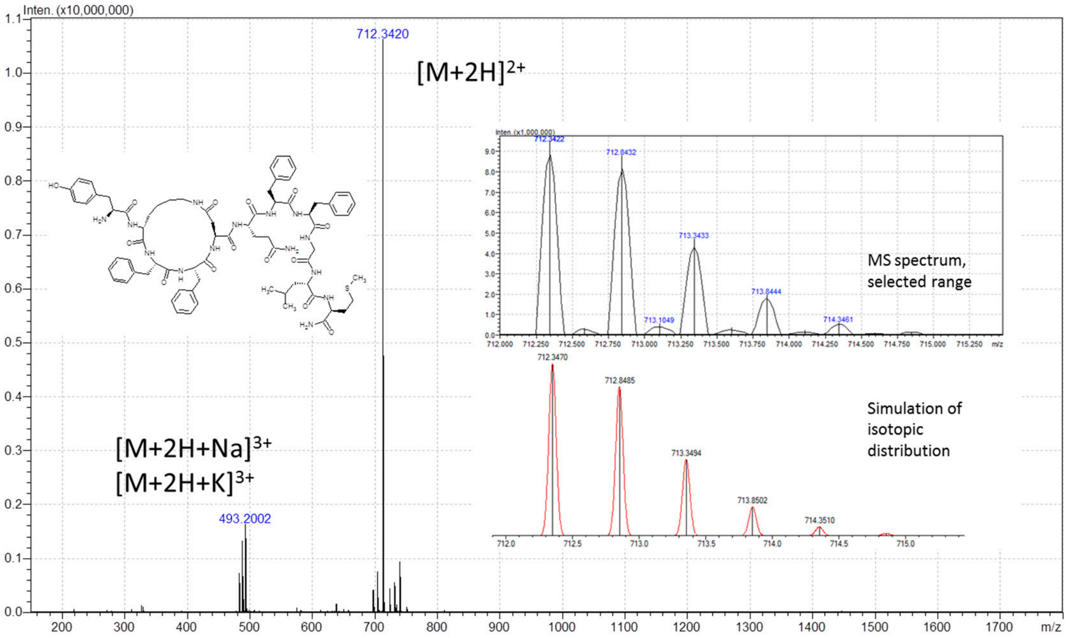

Figure S3. High resolution MS spectrum of peptide H-Tyr-(D-Lys-Phe-Phe-Asp)-Gln-Phe-Phe-Gly-Leu-Met-NH_2 (analog 4). In inset, fragment of the experimental spectrum is compared with the simulated isotopic profile calculated for the expected molecular formula of protonated species $[M+2H]^{2+}$ (bottom panel).

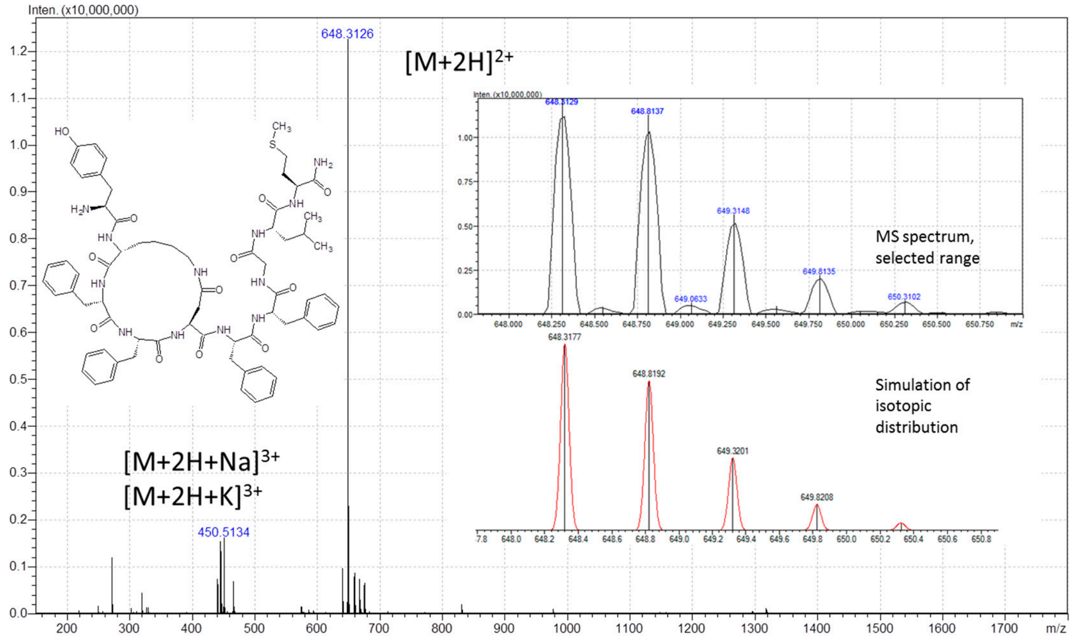

Figure S4. High resolution MS spectrum of peptide H-Tyr-(D-Lys-Phe-Phe-Asp)-Phe-Phe-Gly-Leu-Met-NH_2 (analog 5). In inset, fragment of the experimental spectrum is compared with the simulated isotopic profile calculated for the expected molecular formula of protonated species $[M+2H]^{2+}$ (bottom panel).

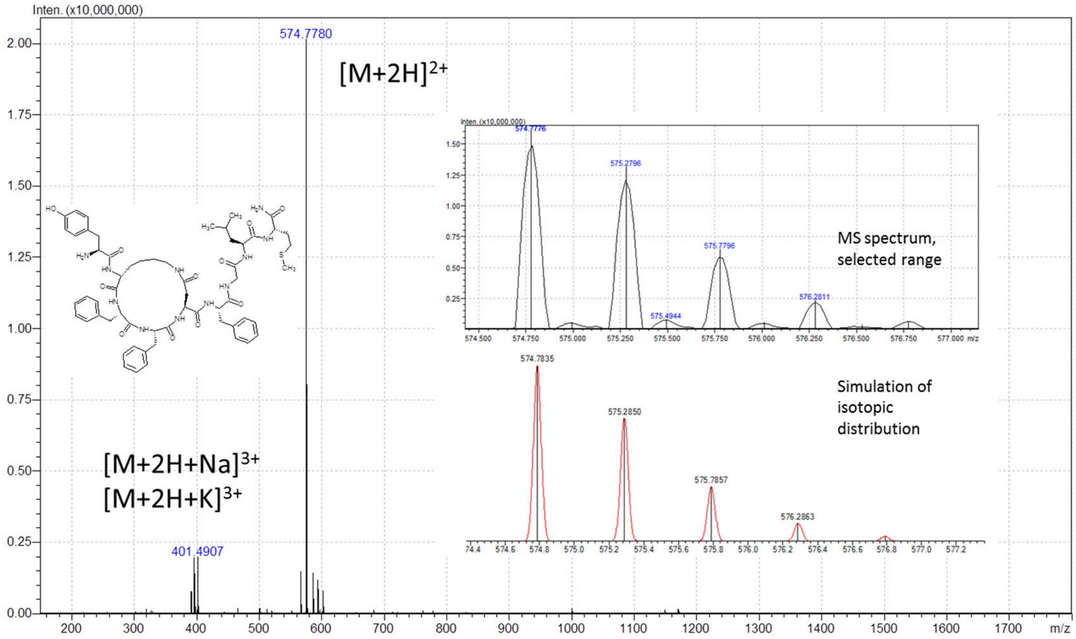

Figure S5. High resolution MS spectrum of peptide H-Tyr-(D-Lys-Phe-Phe-Asp)-Phe-Gly-Leu-Met-NH_2 (analog 6). In inset, fragment of the experimental spectrum is compared with the simulated isotopic profile calculated for the expected molecular formula of protonated species $[M+2H]^{2+}$ (bottom panel).

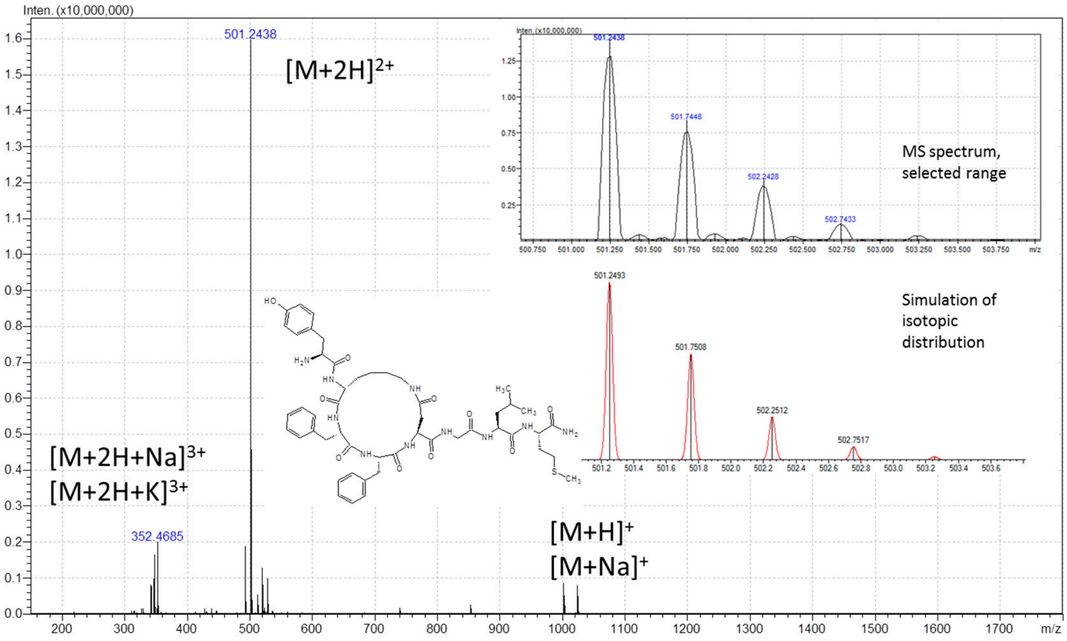

Figure S6. High resolution MS spectrum of peptide H-Tyr-(D-Lys-Phe-Phe-Asp)-Gly-Leu-Met-NH_2 (analog 7). In inset, fragment of the experimental spectrum is compared with the simulated isotopic profile calculated for the expected molecular formula of protonated species $[M+2H]^{2+}$ (bottom panel).

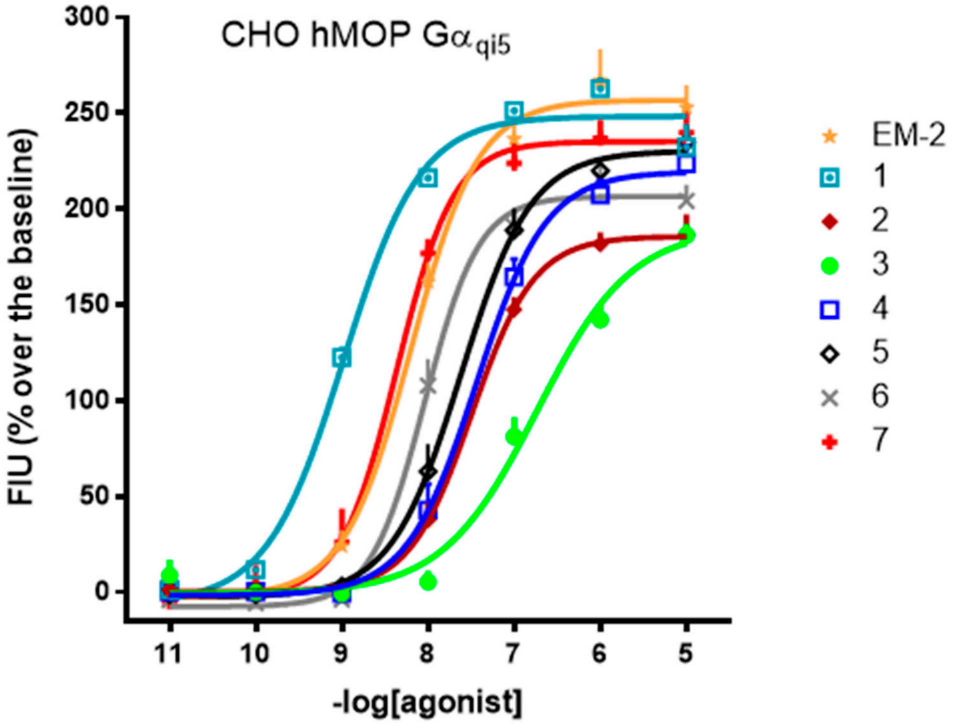
CHO hMOP Gα qi5
FIU (% over the baseline)
300
250
200
150
100
50
0
11
10
9
8
7
6
5
-log[agonist]
EM-2
1
2
3
4
5
6
7

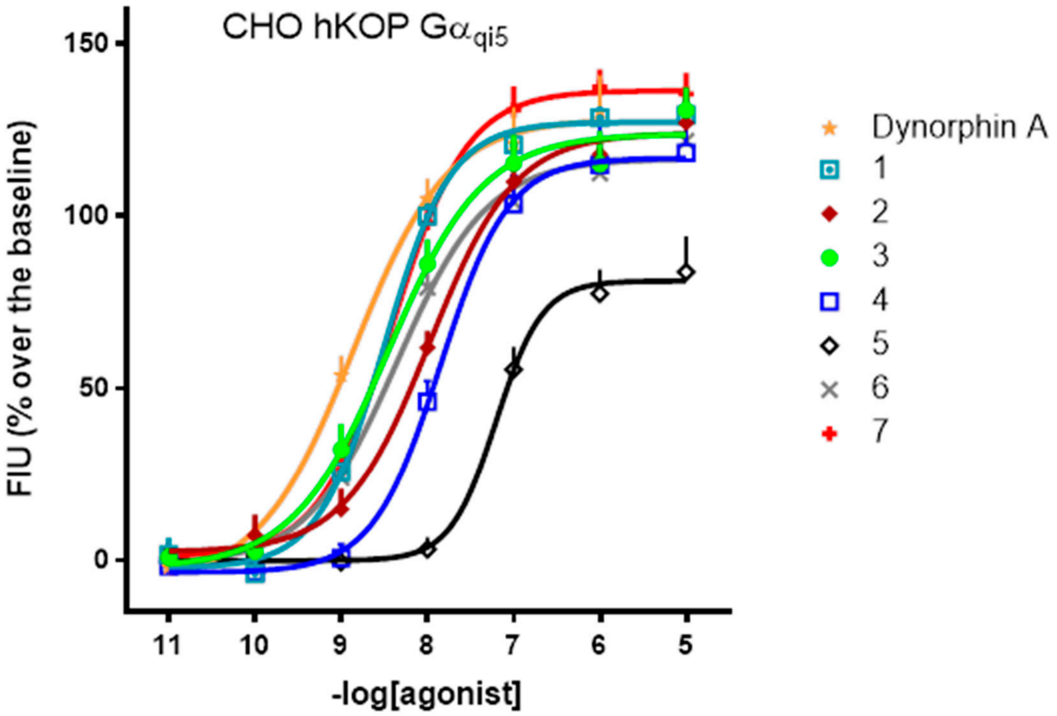
CHO hKOP Gα qi5
FIU (% over the baseline)
150
100
50
0
11
10
9
8
7
6
5
-log[agonist]
Dynorphin A
1
2
3
4
5
6
7

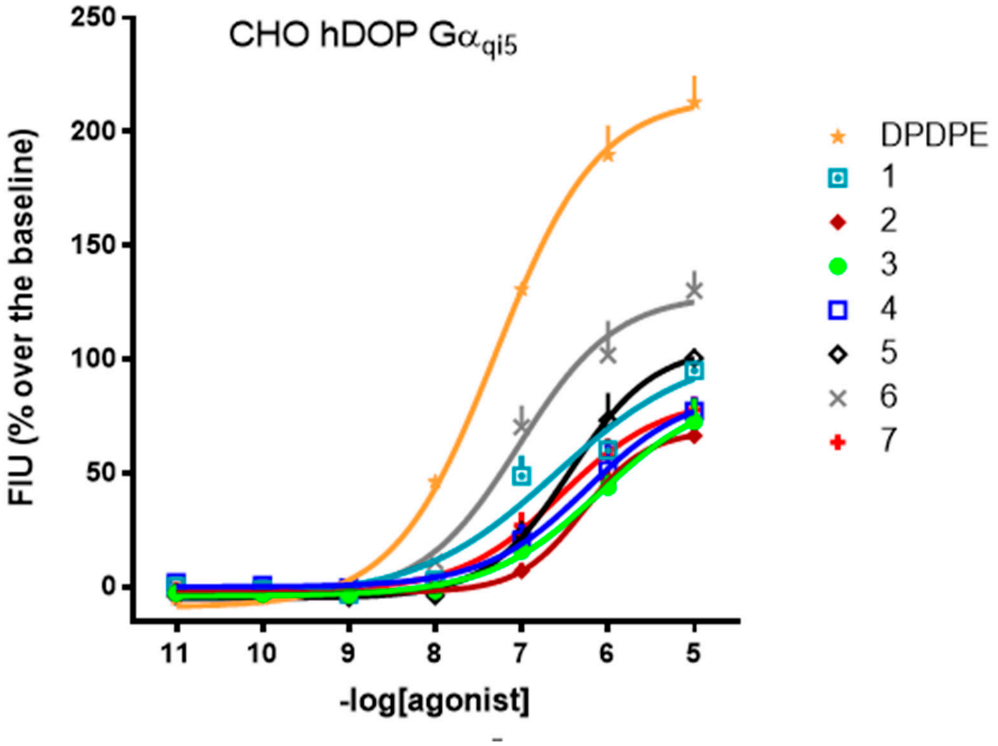

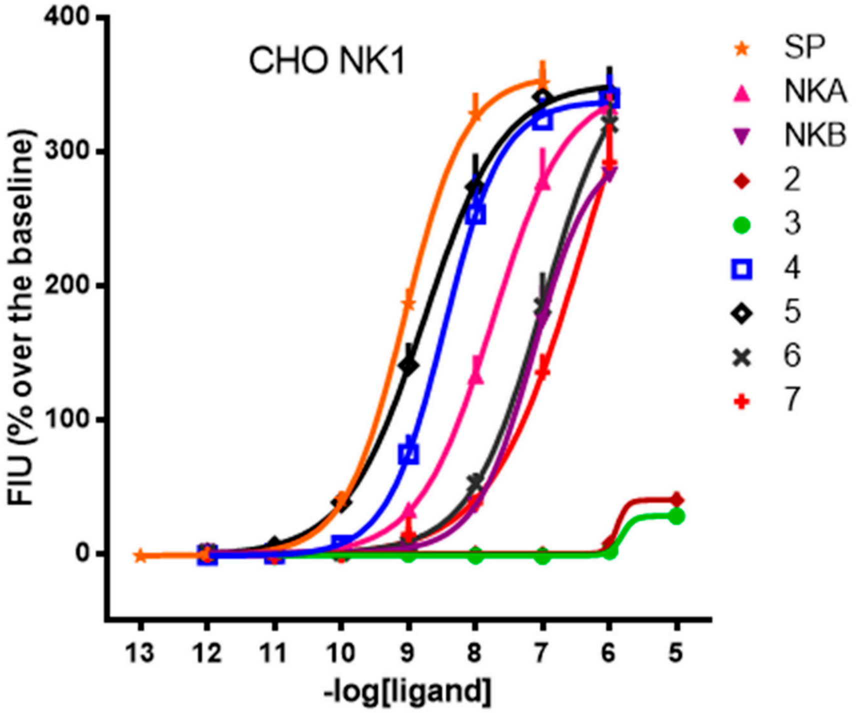

Figure S7. Concentration response curves of EM-2 (1) and analogs 2-7 in calcium mobilization experiments.
